# Rabies Virus RNA in Naturally Infected Vampire Bats, Northeastern Brazil

**DOI:** 10.3201/eid1612.100726

**Published:** 2010-12

**Authors:** Aroldo J.B. Carneiro, Carlos R. Franke, Andreas Stöcker, Flávia dos Santos, José E. Úngar de Sá, Evandro Moraes-Silva, José N.M. Alves, Sebastian Brünink, Victor M. Corman, Christian Drosten, Jan F. Drexler

**Affiliations:** Author affiliations: Federal University of Bahia, Salvador, Brazil (A.J.B. Carneiro, C.R. Franke, A. Stöcker, F. dos Santos);; Central Laboratory of Public Heath, Salvador (J.E. Úngar de Sá);; Agency for Agriculture and Livestock Defence of Bahia, Salvador (E. Moraes-Silva, J.N.M. Alves);; University of Bonn Medical Centre, Bonn, Germany (S. Brünink, V.M. Corman, C. Drosten, J.F. Drexler)

**Keywords:** Rhabdoviridae, lyssavirus, rabies virus, viruses, Brazil, vampire bats, transmission, virus concentrations, letter

**To the Editor:** Rabies is a major zoonotic disease and causes >55,000 human deaths annually worldwide (*1*). The predominant infection route for humans is by canids but zoonotic transmission from bats has been reported (*2**,**3*). Of >1,000 bat species, the only 3 species that feed on blood (*Desmodus rotundus*, *Diphylla ecaudata*, and *Diaemus youngi*) are found exclusively in Latin America (*4*). Rabies outbreaks caused by *D*. *rotundus* vampire bats have resulted in human deaths in Latin America and estimated livestock losses of $6 million annually (*4*).

To study rabies virus (RABV) prevalence and transmission in bat populations, we sampled 199 suborder Microchiroptera bats (mostly from families Phyllostomidae [86.4%] and Molossidae [11.1%]) in Bahia, northeastern Brazil, during 2008–2010. Areas where vampire bat activity or rabid livestock were reported were visited by members of the Bahia State Agency for Agriculture and Livestock Defence to identify bat roosts. All sampling was approved by the Brazilian Institute of the Environment and Natural Renewable Resources.

Bats were caught at roosts by using mist nets, killed with ether, and transported on ice to our laboratory. In accordance with rabies control program policies in Brazil, only vampire bats that were physically impaired (e.g., poor flight ability) or found dead could be sampled.

Thirty milligrams of brain or medulla oblongata per animal was homogenized and purified by using the RNEasy Kit (QIAGEN, Hilden, Germany). RNA was detected by using nested reverse transcription–PCR (RT-PCR) specific for viral nucleoprotein gene (*5*). RABV RNA was detected in 8 (27.6%) of 29 *D. rotundus* bats.

The 8 bats originated from 6 of 9 sampled roosts located in an area of ≈7,200 km^2^. Nucleotide sequencing of PCR amplicons confirmed close phylogenetic relationships with vampire bat RABV (GenBank accession nos. HM171529–HM171536), which is consistent with reported absence of other *Lyssavirus* species in the Americas (*4*). Conventional RABV diagnostic tests (direct immunofluorescent test and infection of suckling mice) confirmed presence of RABV in central nervous system specimens from all 8 bats.

Viruses were quantified by using strain-specific real-time RT-PCR with the One Step RT-PCR Kit (QIAGEN) and primers BRDesrot-Fwd, 5′-CGTACTGATGTGGAAGGGAATTG-3′; BRDesrot-Probe, 5′-FAM-ACAAGGGACCCTACTGTTTCAGAGCATGC-3′-Black Hole Quencher 1; and BRDesrot-Rev, 5′-AAACTCAAGAGAAGGCCAACCA-3′. Absolute quantification was performed by using in vitro–transcribed cRNA for the specific region.

Muscle, interscapular brown fat, tongue, and reproductive, thoracic, abdominal, and retroperitoneal organs from all 8 RABV-positive bats were tested. RNA concentrations were consistently highest in central nervous system specimens (median 10^10.91^ genome copies/g tissue) (Figure A1). Tongue specimens (containing salivary glands) also showed high concentrations (median 10^8.66^ copies/g tissue). High concentrations in heart and lung were compatible with anterograde virus secretion through the vegetative system, similar to that in other mammals (*6*). Not all spleen samples were RABV positive, which suggested no specific involvement of RABV with the lymphatic system. Two of 5 female RABV-positive vampire bats were pregnant at the time of sampling. Virus was detected in both placentas and in 3 of 4 uterus specimens tested (median 10^6.55^ copies/g tissue). However, the 2 fetuses were too immature for analysis. Testicle specimens were available for 2 of 3 male bats; 1 bat was positive (10^6.76^ copies/g tissue).

Modes of RABV transmission and pathobiology in bat populations are unclear. RABV infection at high doses leads to death. High seroprevalence rates in populations of apparently healthy animals suggest that bats may be capable of controlling natural infection, in contrast to other mammals (*6*). Nevertheless, these findings and those of another study (*7*) demonstrated that bats may also die from natural RABV infection. However, because only moribund vampire bats were sampled in our study, the proportion of bats that die of natural infection is unknown.

Although regurgitation of blood for feeding offspring or roost mates was suggested to be a route of infection in vampire bats (*8*), the organ distribution of RABV in our study suggests secretion from salivary glands after spread from the central nervous system, which is compatible with virus transmission in other mammals. Our finding is also consistent with those of a study on tissue distribution of European bat Lyssavirus 2 in *Myotis daubentonii* bats (*9*).

Increased virus concentrations in placentas and reproductive organs suggest vertical transmission, supporting previous findings of RABV in reproductive organs of a deceased *Eidolon helvum* bat (*7*). However, whether similar observations can be made in healthy bats is unknown. Sporadic detection of virus and low virus concentrations in bladder and intestine make RABV transmission by excreta less likely (*7**,**9*). Whether RABV infection was the primary cause of disease in our RABV-positive bats is unknown. Distribution of RABV in organs of moribund vampire bats was similar to that observed in autopsy specimens from humans (*10*). Thus, if we assume that the patterns of organ distribution we observed are representative for free-ranging vampire bats, transmission patterns may be similar to those seen in other mammals.

## Figures and Tables

**Figure A1:**
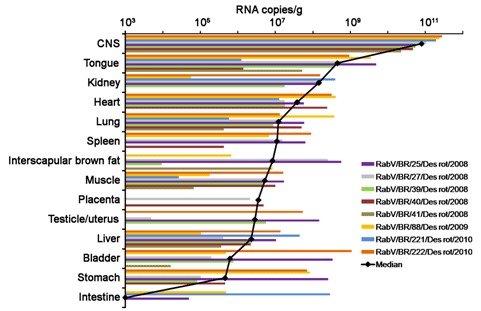
Rabies virus RNA concentrations in organs of vampire bats (*Desmodus rotundus*), northeastern Brazil. CNS, central nervous system.
